# COVID-19 in China: Responses, Challenges and Implications for the Health System

**DOI:** 10.3390/healthcare9010082

**Published:** 2021-01-16

**Authors:** Cunhai Xing, Ruilian Zhang

**Affiliations:** 1 School of Marxism, Hohai University, Nanjing 211100, China; xingch@hhu.edu.cn; 2 Sustainable Minerals Institute, The University of Queensland, Brisbane 4072, Australia

**Keywords:** public health crisis, health system, challenges, COVID-19

## Abstract

A public health crisis is a “touchstone” for testing the ability and capacity of a national health system. In the current era, public health crises are presenting new systematic and cross-border characteristics and uncertainty. The essence of a system for public health crisis governance is the rules administering the stimulus–response chain. The health system generally emphasizes joint participation and communication between different subjects, which may lead to overlap and redundancy as well as a lack of auxiliary support for major public health crisis events. In the context of coronavirus disease 2019 (COVID-19) in China, we track the responses, challenges, and implications of the temporary disruption of the health system and its response to this major public health crisis. We examine local governance capacity, performance in pandemic control, and the coordinated responses to COVID-19. Accordingly, we identify the challenges to the health system, including the imbalance in attention given to medical care versus health care, insufficient grassroots public health efforts and control capacity, and untimely information disclosure. It is strongly suggested that the government improve its cognitive ability and focus more attention on building and strengthening the emergency health system.

## 1. Introduction

The world is facing a great change, driven by a pandemic of a scale not seen for a century. The global spread of the new pandemic in 2020 was the first major “super-accelerator” driving differentiation and new combinations in the global order since the 2008 financial crisis [1,2]. The financial crisis in 2008 was regarded by many international relations scholars as the first serious turning point in the global order since World War II. After 2008, the original global governance system faced the risk of experiencing a “hollowing out of power”. Global governance experts have explored new forms of international practice, and regions have become another important stage for maintaining, coordinating, and managing relations between countries [3,4]. In the 21st century, governance ability at the regional level has gradually emerged as a concern. China has made important contributions to public health system emergency management in response to the coronavirus disease 2019 (COVID-19) pandemic, but there are still areas that could be further improved [5].

A mature public health system and modern governance capacity are important guarantees of national health and socioeconomic development. They are also important supports for responding to major public health emergencies [3]. Governance is a necessary condition if the public health system is to play an effective role. In recent decades, great achievements have been made in China’s public health reform. National nutrition, physical fitness, drinking water, sanitation facilities, disease incidence, and the medical security system have all improved. Life expectancy at birth has increased from 35 years in 1949 to 77 years in 2018 [6]. After China experienced the severe acute respiratory syndrome (SARS) pandemic in 2003, the construction of a national public health emergency management system was put on the agenda. The initial signal of its formation was the “Promulgation of the National General Plan for Public Emergencies” on 8 January 2006 [7]. Public health events such as the SARS and influenza A virus subtype H1N1 (H1N1) outbreaks in 2003 and 2009, respectively, further promoted the reform of China’s public health system, and a series of public health and medical reform regulations were issued. The year 2020 represents the comprehensive end of the fight against poverty, as it was the year China designated for realizing the goal of building a moderately prosperous society. On 29 January 2020, when the first case of COVID-19 was diagnosed in Tibet, this meant that the last mainland area within China’s territory was infected with the virus [8]. The Communist Party of China (CPC) and the government took active measures to mitigate the pandemic, including large-scale quarantines, travel restrictions, and the isolation and monitoring of suspected cases. COVID-19 has been a major test of China’s public health system and governance capacity and illustrates the vital importance of improving major pandemic prevention and control systems along with national public health system emergency management [9]. However, to the best of our knowledge, there is limited research on challenges to the health system in China in the face of COVID-19. The world, especially China, is facing a second wave of COVID-19 cases from overseas. Thus, it is vital to understand what China can learn from the successes and failures of the first wave of COVID-19 so that it can apply these lessons to future public health crisis governance.

This study has two goals: (1) to track the responses of China’s local and central governments to COVID-19 and (2) to identify the challenges faced by the Chinese health system with respect to this public health crisis. These two goals have implications for improving the health system. As its contribution, this research (1) presents the differences in the cognitive abilities of local and central governments when facing public health emergencies and (2) identifies the economic, health care-related, and information disclosure-related challenges to the health system. Four sections follow this introduction. Section 2 introduces the responses of China’s local governments to COVID-19. Section 3 explains the challenges facing the health system in China. Section 4 presents the implications for improving public health system emergency management. Section 5 concludes the paper.

## 2. Responses to COVID-19

### 2.1. Local Governance Capacity and Pandemic Control Performance

Cognitive ability can be defined as a “mental capability that involves the ability to reason, plan, solve problems, think abstractly, comprehend complex ideas, learn quickly and learn from experience” [10]. It incorporates components related to the ability to learn and adapt, and its core is the ability to carry out complex information processing [11]. The cognitive ability of a government affects its response to the trigger of a public health emergency. We argue that during this public health crisis, China’s local governments demonstrated a level of cognitive ability that supports good governance, as illustrated by the examples below [12].

Guangdong and Henan Provinces both demonstrated excellent cognitive ability when facing this public health emergency. Guangdong Province was the first province in China to respond to COVID-19 based on the lessons learned from SARS in 2003. Official information disclosure not only considers people’s emotional comfort but also provides clear professional guidance on pandemic prevention [13]. From the beginning, the timely disclosure of information, together with a significant lead in releasing official news, set the prevention and control of COVID-19 in Guangdong on a different path compared to the SARS experience 17 years prior. The quality of information obtained by citizens was also greatly different. For COVID-19, under new information disclosure modes, events characteristic of the SARS period among the people of Guangdong Province, such as rumors about a strange disease and skyrocketing purchases of white vinegar as an anti-viral treatment, did not occur [14,15]. Instead, the public effectively cooperated with the emergency control measures issued by the government in the face of the pandemic, achieving close cooperation and positive interaction between the government and the public. Clearly, the experience of SARS in Guangdong Province greatly improved the cognitive ability of the provincial government. It allowed unobstructed information exchange and cooperation between the government, disease control authorities, and medical institutions and enabled a very rapid response to the pandemic [16].

The performance of Henan Province in the prevention and control of the pandemic went beyond the expectations of the public. The pandemic prevention measures surprised countless netizens and showed that the Henan government had a strong cognitive ability and governance capacity despite its weak economic strength and large population inflow during the pandemic [17]. Henan Province was aware of the enormous risks associated with the pandemic as early as the end of December, although China’s central government had not announced that COVID-19 had the characteristic of human-to-human transmission. For example, a COVID-19 medical treatment center was designated in Henan, the Zhengzhou shuttle bus route to Wuhan was closed at the end of December, and live poultry sales were banned on 21 January. On 22 January, 130 newly designated COVID-19 treatment hospitals were announced. On 23 January, the government called on Wuhan returnees to report to their village and street neighborhood committees in a timely manner and to isolate at home for 14 days. A press conference on prevention and control was held on 24 January. After local governments realized the severity of the pandemic, their resource allocation and pandemic prevention measures were timely and effective [18]. However, the cognitive abilities of local governments varied.

Wuhan city, also known as the “Chicago of China”, is the capital and largest city of Hubei Province in central China. It has a population of over 11 million and is considered a major industrial and economic center in the region. In December 2019, several cases of the emerging coronavirus were reported in Wuhan. These patients, presenting with pneumonia, were thought to have one of the countless viruses that present the same symptoms [19]. Over the first three weeks of January, Wuhan officials stated that there were only a few dozen confirmed cases, and they downplayed the risk of human transmission. Later, in early and mid-January 2020, more cases started to appear in other provinces due to population movement during the Chinese New Year holiday. On January 23, Wuhan was placed under a strict quarantine. By this time, the situation in Wuhan was totally out of control, and other cities just one hour’s drive away were completely unprepared. Many poor outcomes could have been avoided if people had only been told the truth about the virus before this point [20]. If the Wuhan government had realized the severity of the pandemic and disclosed information to residents early on, Wuhan would have had sufficient capacity to halt the spread of COVID-19 in the initial stages. However, Wuhan’s response was relatively slow, especially in terms of information disclosure. The root cause was its insufficient cognitive ability [19,20].

### 2.2. Central Government-Led Actions to Deal with COVID-19

China’s pandemic prevention and control have fully proven the advantages of collaborative governance. Specific implementation is under the leadership of the CPC, with the government playing the leading role and non-governmental organizations (NGOs), enterprises, the public, and multiple other subjects participating in collaborative governance [1]. This multi-subject approach makes full use of modern scientific and technological means in a grid governance mode. This approach divides urban communities and rural communities under one jurisdiction into unit grids, implements closed management and self-service approaches, and thereby can effectively curb the spread of a pandemic [20,21]. Under the guidance of health and pandemic prevention experts, the participants cooperate to meet goals that cannot be met or realized by a single entity. Notably, although the subjects of pandemic prevention and control are diverse, collaborative governance is always multi-subject collaborative governance under the leadership of the CPC. The leadership of the CPC is the core link maintaining a comprehensive view and the coordination of all parties in the prevention and control of a pandemic situation. Government leadership is an important guarantee of security, order, and resource coordination in pandemic prevention and control, while the participation of communities, NGOs, enterprises, and the public is the social force behind pandemic prevention and control [22].

First, the CPC plays a core leadership role in collaborative governance, and although it has been criticized by scholars and officials in Western countries, it plays a central role in linking organizations at all levels. The advantage of China’s political system is the centralized and unified leadership of the CPC. In the event of an emergency, the CPC takes on the responsibility and role of command over the total situation and coordinates all parties, transforming the advantages of its centralized system into efficient governance. Under the unified leadership of the CPC, the anti-pandemic campaign rapidly mobilized all forces and resources to cope with the pandemic and maximize the efficiency of resource allocation [23].

Second, the government plays a leading role in collaborative governance. When fighting a pandemic, the market mechanism usually fails in resource allocation. When the market is not functioning normally, it becomes even more necessary for the government to lead [24]. The government must set up a data information and transmission system from the central level to the local, community, and grassroots levels to transmit timely and accurate pandemic information and to quickly make scientific decisions [25]. The government should concentrate on the allocation of resources for production, distribution, and use; give full play to the advantages of state-owned enterprises; and motivate enterprises to increase production to ensure the effective, sufficient, and sustainable supply of anti-pandemic resources. At the same time, it is necessary for the state to adapt its fiscal and tax policies to reduce taxes, rents, and other burdens to minimize the pressures on large- and medium-sized enterprises and self-employed households. It is important to increase the reserves of basic living materials available on the market and to decrease people’s costs of living. In response to COVID-19, the government led the socialist system to focus on major events and achieved many miracles: in just 10 days, more than 2000 beds were set up in the Thunder God Mountain (Lei Shen Shan in Chinese) and Fire God Mountain (Huo Shen Shan in Chinese) hospitals. The army sent 1400 medical and nursing personnel to undertake medical treatment tasks in Wuhan’s new pneumonia specialist hospital. In addition, 15 shelter hospitals with a total of 100,000 beds were rapidly built to treat patients with mild cases of the disease, and more than 42,000 medical staff were sent to Hubei Province from provinces, municipalities, and autonomous regions across the country. Most factories quickly resumed the production of medical materials and fully mobilized human, material, financial, scientific, and technological resources to ensure supply [26,27].

Third, social forces play a supporting role in collaborative governance. Previous disaster reduction and prevention events have proven that the extensive participation of social forces achieves remarkable results [28]. The multi-subject agency of NGOs, enterprises, the public, and individuals is the main force coordinating pandemic prevention and control. The power of social forces is reflected in donations of money and goods; a large number of enterprises and individuals provided substantial financial support as well as a large number of oxygen tanks, articles of protective clothing, face masks, and other short-term donations. Numerous volunteers participated in emergency services; for example, the Love Team delivered living materials, and the compulsory mobilization of medical personnel and alumni associations around the country actively organized and coordinated the procurement of protective materials and equipment from overseas [10,11]. These efforts were manifested in the community, and volunteers focused on special groups. A collaborative platform was established to allow more individuals to invest in pandemic prevention and control, taking the Internet as the medium and social media as the carrier to provide rapid information dissemination and communication. In pandemic prevention and control practices, there are also cases of government and social forces working together to build such a platform [16].

## 3. Challenges to the Health System

Although the CPC, the Chinese government, and NGOs made great contributions to slowing the spread of COVID-19, COVID-19 nonetheless became a global pandemic. What hindered the government from achieving a more integrated and effective public health system to fight the pandemic?

First, governments are faced with a trade-off between economic development and public health improvement [5,6]. In the long term, as a critical component of human capital, health is an important engine of economic development. Improving health has a positive impact on both micro-labor productivity and macro-economic growth. However, in the short term, increases in public health expenditure will produce a crowding-out effect. On the one hand, private health investment will be squeezed out, which will increase the financial pressure on the government [11]. On the other hand, other public infrastructure investments will also be crowded out. How government officials choose between economic development and public health improvement depends on the method of assessment and election. In the political competition system, gross domestic product (GDP) is the target used for performance evaluation, and thus GDP becomes the core goal of government officials, and public health may become a victim. In recent years, the central government has emphasized that the assessment of GDP should play a smaller role, and political competition centered on economic growth has undergone a series of adjustments [9]. However, as of 2017, China’s government health expenditures as a proportion of GDP have been hovering at a low level, basically controlled at approximately 8%, which is far lower than the level of major developed countries and even that of some developing countries in the same period. Therefore, local governments should not underestimate investment in public health, either from the “quasi-public goods” perspective of public health resources or from the perspective of the “healthy China” development strategy.

Second, China’s health system has paid more attention to medical care than to health care, and its service provision is fragmented. Hospitals account for 54% of China’s total health expenditure, compared with an average of 38% in countries belonging to the Organisation for Economic Co-operation and Development (OECD). The ten-year medical reform in China from 2009 to the present focused on the quality of medical services but neglected that of public health [19]. Public health is an important factor influencing health, affecting not only the health of individuals but also the overall health of society. In China, over the ten-year period of health care reform, investments have been made in the direct reporting system. Although the system played a positive role in preventing the spread of H1N1, it was virtually useless in the early stage of the COVID-19 outbreak. The proportion of health personnel in disease control institutions in China has decreased from 2.53% in 2009 to 1.53% at present. Although the “healthy China” development strategy clearly defines a guiding ideology of public health with prevention as the priority, the actual situation shows that there is a gap between the ideology and reality in terms of salary distribution and investment guarantees [22,23]. Public health is a form of public welfare, which is different from the paid medical services of hospitals; thus, many corresponding mechanisms are difficult to implement.

Third, problems with the functions, capabilities, and powers of the public health system have resulted in the fragmentation of public health governance. China’s Centre for Disease Control (CDC) has a vertical management structure in terms of business guidance. However, administrative leadership is horizontal, and the CDC has no personnel rights at the higher level or financial rights at the lower level; thus, it can only arrange work and is not responsible for funds and wages, resulting in low management efficiency and an inability to quickly respond to public emergencies [29]. The medical and health industry involves more than ten government departments, all of which are committed to achieving their own institutional goals. Therefore, there are problems coordinating health institutions at all levels and a countrywide lack of effective communication mechanisms; such institutions even compete with and exclude each other, increasing the overall cost of disease prevention and control and hindering the process of medical and health reform. In addition, the emergency mechanism of major public health events requires managers to have a biomedical background. Under the current public health system, with inconsistent powers and responsibilities, the managers of local health and disease control departments are mainly appointed by local governments and are mostly non-professionals [30]. Therefore, among public health managers, there is insufficient relevant knowledge or attention on the role of professionals. After the SARS pandemic ended in 2003, the World Health Organization organized a team of experts from China and other countries to track the origin and early transmission route of the SARS virus. At that time, seven of the eight experts in the international expert group had a background in veterinary medicine or animal health, while only one of the six experts in China had such a background.

Fourth, China’s grassroots public health prevention and control capacity is insufficient. The grassroots public health system is the key to dealing with public health emergencies because of its special position on the “front lines” [2,3]. Grassroots and village committees have obvious information network advantages, and they can accurately and quickly identify various neighborhood problems. Although the total number of medical and health personnel in China has increased in the past decade, it is still difficult for primary health institutions and poor rural areas to attract and retain qualified medical personnel [13]. The proportion of primary health personnel on health teams decreased from 40% in 2009 to 36% in 2013. Moreover, a major difference between the service quality of primary health institutions and hospitals in China discourages patients from visiting primary medical institutions. Most grassroots health workers have a low educational level, lack diagnostic ability, and have limited knowledge of infectious disease response; as a result, there is public distrust of their ability, knowledge, and information [16]. However, despite the weak prevention and control ability of grassroots public health institutions, they are the main component of the overall medical and health system. Therefore, it is necessary to strengthen the grassroots prevention and control capacity in rural areas and communities and to organize this first line of defense [22].

Finally, information disclosure is not timely [1]. Due to the government’s inadequate preparation for health education and the lack of health information dissemination channels, the public cannot take effective measures in the face of public health emergencies. In the early stage of the pandemic, public health knowledge was very limited [3]. The government issued a statement but lacked in-depth efforts in the community, such as publicizing knowledge about the new coronavirus and providing guidance for isolation and disinfection practices. Even in the government’s press conferences and the speeches of professionals, there were contradictions and omissions in refuting rumors [28].

## 4. Implications for Improving Public Health System Emergency Management

The COVID-19 outbreak has seriously affected people’s safety and national security. It is essential to prioritize national strategies to take prevention and control as important tasks, defuse major risks, and enhance the ability to prevent and control public health events as an important part of the national governance system and governance capacity [31]. It is never too late to take full advantage of this experience for pandemic prevention and major risk control in the field of health. Furthermore, there is an urgent need to improve public health system emergency management [32].

### 4.1. Improve the Cognitive Ability of the Government

First, the government’s awareness of the risk of public health emergencies should be improved. The governance capacity of the government at all levels is an important factor that is currently preventing the public health system from fully realizing maximum prevention and control [3]. The comprehensive governance capacity of the government is mainly limited by its cognitive ability, as it is difficult for the government to give full play to its strong executive powers given its insufficient cognitive ability. Therefore, to improve governance capacity in the field of public health, the most urgent task is to enhance the government’s cognitive ability [7]. This can be done by adjusting the assessment system for local officials, balancing economic growth goals with public health objectives, clarifying the rights and responsibilities of public health departments, and lessening the fragmentation of public health system management. At the same time, the government should take practical measures to strengthen communication with universities and professional institutions, promote the transformation of the governance mode, and realize governance modernization in the field of public health [9,10]. In addition, the smooth and transparent communication of information is necessary in public governance. The modern public health system and public governance need governments at all levels to make full use of modern media to strengthen the dissemination and communication of health information [21]. The government should strengthen the legal construction of information disclosure, enhance the transparency of information disclosure, and give full play to the role of new media in positive publicity. The government should also follow developments in science and technology to achieve better results by enriching information disclosure channels. Although the economic losses caused by the pandemic are inevitable, if provincial and municipal governments can actively respond, they can still improve the public health situation in all provinces and cities in China and accelerate economic growth during the long recovery process [33].

### 4.2. Strengthen the Health Care System Rather than the Medical System

Second, the primary health care system should be strengthened. Because China’s public health system places too much emphasis on medical treatment and ignores the construction of the grassroots public health system, the primary health care system lacks medical capacity and credibility, and staff lack experience and the knowledge to deal with infectious diseases [4]. These problems limit the full use of medical and health resources. Therefore, in future medical reforms, attention should be paid to the primary health care system [6]. It is important to more quickly activate and promote the implementation of the hierarchical treatment system [10]. The public health system not only aims to expand human health knowledge and promote technological progress but also is an industrial and commercial system with medical products and the supply of services [11]. Science, industry, and systems are each embedded in the others. Any problem in one dimension may make it difficult to operate the public health system effectively. Therefore, in the face of the crisis in the public health system, scientific, and technological knowledge, the market and the national system should be jointly constructed. To protect vulnerable groups is to protect society, and a consensus should be reached on this point in society beyond the scientific consensus on the importance of public health [14].

### 4.3. Enhance the Use of Information Technology in Health Systems

Third, it is essential to use new information technology to build an intelligent public health emergency management system [34]. Public health security is an important part of national security and a common challenge across humankind. The scientific management of public health safety risks must rely on modern information technology. China’s public health emergency management system has built a certain foundation, especially the establishment of emergency management departments in the institutional reform promulgated by the State Council in 2018 to further centralize and integrate decentralized emergency resources and emergency management agencies [4]. In this context, China’s public health emergency management system is advancing in a scientific, optimized, coordinated, and efficient direction. It is important to accelerate the construction of emergency management databases for major pandemic situations, and integrate emergency management, health, industry, and information data [18,19]. The government should improve its disease transmission models, grasp the trends and risks in disease spread, improve its ability to rapidly analyze and provide information on major public health emergencies, and promote intelligent emergency decision-making [30]. In particular, the application of new technology for major pandemic prevention and control can help with five aspects of a national public health emergency management system: the construction of a legal guarantee of public health, improvements in disease prevention and control, major pandemic treatment, medical insurance and rescue, and unified emergency material support systems [25].

### 4.4. Strengthen Health Protection Laws and Regulations

Fourth, there is an urgent need to strengthen and improve the construction of relevant laws and regulations in the field of public health. At present, China’s legal system related to the public health system needs to be improved, and the legislation and support systems for major pandemic prevention and control are incomplete [8]. For example, the “Wildlife Protection Law”, the “Regulations on the Implementation of Terrestrial Wildlife Protection”, and other laws and regulations are not sufficiently clear in terms of prohibiting and restricting the trafficking of edible wild animals. In Article 330 of the “Criminal Law”, there is a loophole in the imprisonment term stipulated for the crime of impeding the prevention and control of infectious diseases [6]. For the “dangerous crime” associated with the risk of spreading a disease and the “actual harm crime” of causing the spread of infectious diseases, offenders are to “be sentenced to fixed-term imprisonment of not more than three years or criminal detention”. The content, scope, and consequences of violating the two laws are obviously unbalanced in terms of risks within the rule of law. Therefore, there is an urgent need to comprehensively strengthen and improve the construction of laws, regulations, and plans in the field of public health systems [11,13]. At the same time, the government should meet the requirements of scientific disease prevention and control, precisely implement policies, help other countries in a similar position, and gradually improve the response system for public health emergencies such as sudden acute infectious disease pandemics [9]. In addition, the government should popularize the laws and regulations on infectious disease prevention and control and enhance public awareness of public health risk prevention and control. Doing so will guide citizens to actively fulfil their obligations in regard to pandemic prevention and control and improve their legal awareness [19].

### 4.5. Improve the Medical Insurance and Relief System and the Emergency Material Support System

Fifth, the government should improve the medical insurance and relief system for major diseases. Major disease relief and insurance systems are important supports for major pandemic prevention and control and national public health emergency management systems [32]. This can be done in two ways. The government should first improve the emergency medical assistance system and, second, establish a medical insurance system for major diseases to ensure that medical institutions at all levels can first treat and then charge in cases of emergency, such as pandemic outbreaks. The government should coordinate the use of public health service funds and basic medical insurance funds [23].

Sixth, the government should improve the unified emergency material support system. China has a vast territory, abundant resources, and a large population. There are many factors that affect and endanger public security [35], and all kinds of potential accidents and safety risks are intertwined, overlap, and frequently occur. Therefore, a sound and unified emergency material support system should not be overlooked. Such a system is expected to overcome many barriers between government departments, between government and enterprises, and between regions to unify multiple links and elements and to save valuable time in emergency response at critical moments [29]. First, the government should improve the emergency material security system for major public health crises, improve cross-departmental and cross-regional prevention activities, and control material supply mechanisms. Second, the government should establish a unified national emergency material production and supply system and optimize the guaranteed production capacity and regional layout of important emergency materials [22,25]. To respond to possible shortages in material supplies, a centralized production scheduling mechanism should be established to maximize the efforts of production enterprises. Third, it is necessary to improve the social donation system and the distribution system for donated materials and to improve material distribution management and information transparency [5].

### 4.6. Reform the Disease Prevention and Control System

Seventh, it is vital to reform and improve the disease prevention and control system [1]. A working mechanism and a mode of close integration and effective connection between disease prevention and control institutions and medical institutions have not yet been established. Such early cross-infection in a pandemic can be prevented with a seamless connection between the CDC diagnosis and hospital treatment [36]. Synergy between scientific research, disease control, and clinical treatment is not sufficient. Data sharing and transformation application channels are not yet reliable, and the abilities to transform scientific and technological innovations into disease prevention and control achievements and to conduct independent research and development into therapeutic drugs are still quite weak [37,38]. Disease control institutions at all levels in China are inactive; they lack strong abilities and have insufficient motivation. Investments in infectious disease medical institutions and the strategic reserve of medical materials are also lacking, and public health talent is in short supply [18]. The public health field lacks technical and skilled personnel. Therefore, there is an urgent need to reform and improve the disease prevention and control system. Governments should improve their capacity to guarantee public health security, health supervision, maternal and child health care, and public health information systems, and they should also improve disease control. The government should improve the mechanism for effectively coordinating clinical treatment, scientific research, and disease control, and it should also improve the collaborative mechanism for research, assessment, decision-making, prevention, and the control of major public health risks [16]. The government should promote a combination of medical care, prevention, and effective connections between public health services and medical services; innovate mechanisms for optimizing the allocation of public health resources; and strengthen the construction of grassroots prevention and control capacity. It is necessary to strengthen public health personnel teams, including the planning and training of administrative personnel, administrative law enforcement personnel, and technical personnel at all levels, mainly based on the aspects of disease prevention and control, health emergencies, education and training, and scientific research; it is also necessary to continue to strengthen the training of general practitioners [16,19].

## 5. Conclusions

COVID-19 not only threatens human health but also has had a major adverse effect on social development. In fighting the pandemic and protecting the lives of citizens, medical workers, biomedical experts, and relevant pandemic prevention units have paid a heavy price. The loopholes in the public health and pandemic prevention systems have been highlighted. The government should pay close attention to these issues and increase investment, continuously improve the urban public health system, and implement coping strategies for public health emergencies. In the face of this unexpected new coronavirus, the Chinese government and people made great sacrifices to prevent and control the first wave of the pandemic; China effectively controlled the spread of the pandemic, explored effective prevention and control measures for travel, and accumulated rich experience in urban health management. At present, COVID-19 has spread to more than 100 countries and regions. The World Health Organization has upgraded COVID-19 to a global pandemic, and it has had a serious impact on people’s lives and health as well as social and economic development worldwide. Many countries failed to achieve effective prevention and control in the early stage of the pandemic, leading to the rapid spread of the disease. In facing the second wave of COVID-19, it is important to pursue the correct path to save lives and to enable the economy to recover by reviewing possible improvements to the health system. We believe that the cost of controlling the pandemic is worthy of discussion. Strict quarantine successfully and efficiently contained the spread of the pandemic, but at an enormous cost to small businesses and many vulnerable populations. People’s privacy might also be sacrificed in the process of tracking this disease. Therefore, it is crucial to find the optimal balance to control the pandemic without major economic or personal sacrifices.

## Data Availability

Not applicable.

## References

[1] Zhang J., Zhang R. (2020). COVID-19 in China: Power, Transparency and Governance in Public Health Crisis. Healthcare.

[2] Abrams E.M., Szefler S.J. (2020). COVID-19 and the impact of social determinants of health. Lancet Respir. Med..

[3] Blair R.A., Morse B.S., Tsai L.L. (2017). Public health and public trust: Survey evidence from the Ebola Virus Disease epidemic in Liberia. Soc. Sci. Med..

[4] Bollyky T.J., Templin T., Cohen M., Schoder D., Dieleman J.L., Wigley S. (2019). The relationships between democratic experience, adult health, and cause specific mortality in 170 countries between 1980 and 2016: An observational analysis. Lancet.

[5] Ahmed F., Ahmed N., Pissarides C., Stiglitz J. (2020). Why inequality could spread COVID-19. Lancet Public Health.

[6] Anner M. (2020). Abandoned? The Impact of Covid-19 on workers and businesses at the bottom of global garment supply chains. Center for Global Workers’ Rights Research Report.

[7] Yip W., Hsiao W.C. (2008). The Chinese Health System at a Crossroads. Health Aff..

[8] Yip W., Hsiao W.C. (2015). What Drove the Cycles of Chinese Health System Reforms?. Health Syst. Reform.

[9] Chan J.F.-W., Yuan S., Zhang A.J., Poon V.K.-M., Chan C.C.-S., Lee A.C.-Y., Fan Z., Li C., Liang R., Cao J. (2020). Surgical mask partition reduces the risk of non-contact transmission in a golden Syrian hamster model for Coronavirus Disease 2019 (COVID-19). Clin. Infect. Dis..

[10] Gottfredson L.S. (1997). Mainstream science on intelligence: An editorial with 52 signatories, history, and bibliography. Intelligence.

[11] Ones D.S., Viswesvaran C., Dilchert S., Evers A., Anderson N., Voskuijl O. (2017). Cognitive Ability in Personnel Selection Decisions. The Blackwell Handbook of Personnel Selection.

[12] Brixi H., Mu Y., Targa B., Hipgrave D. (2011). Equity and Public Governance in Health System Reform: Challenges and Opportunities for China.

[13] Zhou X.D., Li L., Hesketh T. (2014). Health system reform in rural China: Voices of healthworkers and service-users. Soc. Sci. Med..

[14] Tian H., Liu Y., Li Y., Wu C.-H., Chen B., Kraemer M.U.G., Li B., Cai J., Xu B., Yang Q. (2020). An investigation of transmission control measures during the first 50 days of the COVID-19 epidemic in China. Science.

[15] Gianfredi V., Balzarini F., Gola M., Mangano S., Carpagnano L.F., Colucci M.E., Gentile L., Piscitelli A., Quattrone F., Scuri S. (2019). Leadership in Public Health: Opportunities for Young Generations within Scientific Associations and the Experience of the “Academy of Young Leaders”. Front. Public Health.

[16] Hipgrave D., Guo S., Mu Y., Guo Y., Yan F., Scherpbier R., Brixi H. (2012). Chinese-Style Decentralization and Health System Reform. PLoS Med..

[17] Chau T.H., Wan K.M. (2020). Pour (Tear) Gas on Fire? Violent Confrontations and Anti-Government Backlash in Hong Kong. https://ssrn.com/abstract=3557130.

[18] Cheng V.C.-C., Wong S.-C., Chuang V.W.-M., So S.Y.-C., Chen J.H.-K., Sridhar S., To K.K.-W., Chan J.F.-W., Hung I.F.-N., Ho P.-L. (2020). The role of community-wide wearing of face mask for control of coronavirus disease 2019 (COVID-19) epidemic due to SARS-CoV-2. J. Infect..

[19] Burki T. (2020). China’s successful control of COVID-19. Lancet Infect. Dis..

[20] Petrelli F., Grappasonni I., Peroni A., Kracmarova L., Scuri S. (2018). Survey about the potential effects of economic downturn on alcohol consumption, smoking and quality of life in a sample of Central Italy population. Acta Bio Med. Atenei Parm..

[21] Yu X., Li C., Shi Y., Yu M. (2010). Pharmaceutical supply chain in China: Current issues and implications for health system reform. Health Policy.

[22] Heena S., Hunny S. (2020). Role of social media during the COVID-19 pandemic: Beneficial, destructive, or reconstructive?. Int. J. Acad. Med..

[23] Ho L.K.K. (2020). Legitimization & De-legitimization of Police. In British Colonial & Chinese SAR Hong Kong. J. Inter-Reg. Stud. Reg. Glob. Perspect..

[24] Kraemer M.U.G., Yang C.-H., Gutierrez B., Wu C.-H., Klein B., Pigott D.M., du Plessis L., Faria N.R., Li R., Open COVID-19 Data Working Group (2020). The effect of human mobility and control measures on the COVID-19 epidemic in China. Science.

[25] Hopman J., Allegranzi B., Mehtar S. (2020). Managing COVID-19 in Low- and Middle-Income Countries. JAMA.

[26] Li X., Lu J., Hu S., Cheng K., De Maeseneer J., Meng Q., Mossialos E., Xu D.R., Yip W., Zhang H. (2017). The primary health-care system in China. Lancet.

[27] Mo Y., Deng L., Zhang L., Lang Q., Liao C., Wang N., Qin M., Huang H. (2020). Work stress among Chinese nurses to support Wuhan in fighting against COVID-19 epidemic. J. Nurs. Manag..

[28] Pan A., Liu L., Wang C., Guo H., Hao X., Wang Q., Huang J., He N., Yu H., Lin X. (2020). Association of Public Health Interventions With the Epidemiology of the COVID-19 Outbreak in Wuhan, China. JAMA.

[29] Sun Z., Cheng X., Zhang R., Yang B. (2020). Factors Influencing Rumour Re-Spreading in a Public Health Crisis by the Middle-Aged and Elderly Populations. Int. J. Environ. Res. Public Health.

[30] Yuan B., Jian W., He L., Wang B., Balabanova D. (2017). The role of health system governance in strengthening the rural health insurance system in China. Int. J. Equity Health.

[31] Sun Z., Yang B., Zhang R., Cheng X. (2020). Influencing Factors of Understanding COVID-19 Risks and Coping Behaviors among the Elderly Population. Int. J. Environ. Res. Public Health.

[32] Tan W., Hao F., McIntyre R.S., Jiang L., Jiang X., Zhang L., Zhao X., Zou Y., Hu Y., Luo X. (2020). Is returning to work during the COVID-19 pandemic stressful? A study on immediate mental health status and psychoneuroimmunity prevention measures of Chinese workforce. Brain Behav. Immun..

[33] Cowling B.J., Ali S.T., Ng T.W., Tsang T.K., Li J.C., Fong M.W., Liao Q., Kwan M.Y.W., Lee S.L., Chiu S.S. (2020). Impact assessment of non-pharmaceutical interventions against coronavirus disease 2019 and influenza in Hong Kong: An observational study. Lancet Public Health.

[34] Dionne K.Y. (2011). The role of executive time horizons in state response to AIDS in Africa. Comp. Political Stud..

[35] Eimer T., Lütz S. (2010). Developmental states, civil society, and public health: Patent regulation for HIV/AIDS pharmaceuticals in India and Brazil. Regul. Gov..

[36] Block P., Hoffman M., Raabe I.J., Dowd J.B., Rahal C., Kashyap R., Mills M.C. (2020). Social network-based distancing strategies to flatten the COVID-19 curve in a post-lockdown world. Nat. Hum. Behav..

[37] Ahmad A.R., Murad H.R. (2020). The Impact of Social Media on Panic during the COVID-19 Pandemic in Iraqi Kurdistan: Online Questionnaire Study. J. Med. Internet Res..

[38] Wang Cuiyan Pan R., Wan X., Tan Y., Xu L., Ho C.S., Ho R.C. (2020). Immediate Psychological Responses and Associated Factors during the Initial Stage of the 2019 Coronavirus Disease (COVID-19) Epidemic among the General Population in China. Int. J. Environ. Res. Public Health.

